# The ecology–evolution continuum and the origin of life

**DOI:** 10.1098/rsif.2023.0346

**Published:** 2023-11-01

**Authors:** David A. Baum, Zhen Peng, Emily Dolson, Eric Smith, Alex M. Plum, Praful Gagrani

**Affiliations:** ^1^ Wisconsin Institute for Discovery, University of Wisconsin, Madison, WI 53705, USA; ^2^ Department of Botany, University of Wisconsin, Madison, WI 53706, USA; ^3^ Department of Bacteriology, University of Wisconsin, Madison, WI 53706, USA; ^4^ Department of Geoscience, University of Wisconsin, Madison, WI 53706, USA; ^5^ Department of Computer Science and Engineering, Michigan State University, East Lansing, MI 48824, USA; ^6^ Ecology, Evolution and Behavior, Michigan State University, East Lansing, MI 48824, USA; ^7^ Department of Biology, Georgia Institute of Technology, Atlanta, GA 30332, USA; ^8^ Earth-Life Science Institute, Tokyo Institute of Technology, Tokyo 152-8550, Japan; ^9^ Santa Fe Institute, Santa Fe, NM 87501, USA; ^10^ Department of Physics, University of California, San Diego, CA 92093, USA

**Keywords:** autocatalysis, chemical reaction networks, ecology, evolution, origin of life, succession

## Abstract

Prior research on evolutionary mechanisms during the origin of life has mainly assumed the existence of populations of discrete entities with information encoded in genetic polymers. Recent theoretical advances in autocatalytic chemical ecology establish a broader evolutionary framework that allows for adaptive complexification prior to the emergence of bounded individuals or genetic encoding. This framework establishes the formal equivalence of cells, ecosystems and certain localized chemical reaction systems as autocatalytic chemical ecosystems (ACEs): food-driven (open) systems that can grow due to the action of autocatalytic cycles (ACs). When ACEs are organized in meta-ecosystems, whether they be populations of cells or sets of chemically similar environmental patches, evolution, defined as change in AC frequency over time, can occur. In cases where ACs are enriched because they enhance ACE persistence or dispersal ability, evolution is adaptive and can build complexity. In particular, adaptive evolution can explain the emergence of self-bounded units (e.g. protocells) and genetic inheritance mechanisms. Recognizing the continuity between ecological and evolutionary change through the lens of autocatalytic chemical ecology suggests that the origin of life should be seen as a general and predictable outcome of driven chemical ecosystems rather than a phenomenon requiring specific, rare conditions.

## 1. Introduction

Explaining the origin of life requires that we identify a system that was simple enough to arise spontaneously, yet already endowed with the capacity to evolve adaptively and become more complex over time. In modern biology, complexification exploits natural selection, which acts on organismal features encoded in nucleic acid polymers. However, genetic heredity seems too complicated to have arisen without a pre-existing adaptive process. This has posed a persistent problem that has held back the origin-of-life field for many years.

In this paper, we propose that Darwinian evolution is not a discretely delimited mechanism of change but one member of a broader class of autocatalytic chemical ecosystem (ACE) processes. ACE processes are characterized by self-amplifying subsystems called autocatalytic cycles (ACs) that include not only the life cycles of genes and organisms but also motifs that are abundant within abiotic chemical reaction networks. Importantly, because ACs tend to persist once activated (given sufficient flux of food/energy), they serve as a basic unit of memory or inheritance. It can be shown that superficially different ACEs, for example cells, ecosystems and certain localized chemical reaction systems, can all be described using the same formalism.

The first section of this paper reviews prior work on the origin of life, with a particular focus on the emergence of evolvable chemical systems. This section serves to identify the core problem: developing a general description of adaptive evolution that applies to diverse kinds of chemical, biological and population genetic change. The remainder of the paper describes and expands upon recent work in chemical ecosystem theory to explain the emergence of adaptive evolution without cellular encapsulation or genetic encoding, as well as the subsequent origin of cells and genes. We do this by laying out the parallels between a living cell (or organism) and a local ecosystem and between a population of cells/organisms and a meta-ecosystem of interconnected local ecosystems. This implies that both ecological change (e.g. ecological succession) and Darwinian evolution can be seen as formally equivalent in that each entails changes in the frequency of ACs in a meta-ecosystem. We then tie this insight to the origin of life and explore the two main differences between ecological change and Darwinian evolution, namely compartmentalization and genetics, and describe how prebiotic chemical processes might have bridged this apparent gap by gradually becoming more evolution-like and less ecology-like over time. We end by proposing that the conception of cells as chemical ecosystems provides a powerful new framework for guiding both theoretical and empirical studies of the origins of life.

## 2. On the origin of evolution: the state of play

As well articulated by Oono [1], life as we know it today is too complex to have originated directly from non-life but must be the product of a history in which a relatively simple system originated de novo and then accumulated complexity. In modern biology, Darwinian natural selection is sufficient to explain complexification [2], but this mechanism depends on gene-based inheritance systems which are, themselves, very complex. Thus, understanding the origin of life, whether viewed as a historical event on the early Earth or a general phenomenon as might occur elsewhere in the Universe, requires that we identify systems simple enough to arise spontaneously that can nonetheless accumulate complexity.

The molecular biology revolution of the 1950s and 1960s [3] showed that cellular growth/division and genetic inheritance, which are required for Darwinian evolution, require a sophisticated interplay of three interdependent polymer classes, proteins, RNA and DNA, that could not plausibly have emerged spontaneously. The discovery that RNA molecules can both encode information like DNA and have catalytic activities like proteins suggested a solution, namely that evolvable populations of self-replicating RNAs emerged spontaneously and evolved adaptively, with protein and DNA chemistry being added much later [4]. The original RNA World models imagined individual, naked self-replicating molecules [5,6]. However, following the insight that replicating polymers cannot persist if they are longer than a threshold length (the error threshold, set by the accuracy of template-mediated replication) [7,8], most RNA World models shifted to assuming that the first evolving systems were sets of cooperating RNAs enclosed in protocells [9–11]. These models assume the spontaneous appearance of a protocell that happened to be endowed with a set of functional RNA molecules that could collectively replicate, acquire food and energy, and grow and divide. However, many researchers have questioned whether such evolvable protocells are simple enough to arise spontaneously [12,13].

RNA World models have appealed, at least in part, because the mechanism of evolution is familiar, being based on heritable information encoded in nucleic acid molecules. However, the geological rarity and instability of RNA on the early Earth [14] has led several chemists to propose that the first evolving systems were not polymers at all, but autocatalytic sets of chemical reactions, i.e. metabolisms [15–17]. Such metabolism-first models are chemically plausible, but often lack a clear theory of evolutionary change [12,18]. Several models of ‘chemical evolution’ have focused on polymers and template-mediated replication, which reintroduces some of the challenges of RNA World models [19,20].

The peptide network model by Kauffman [21] spawned a rich body of theory focused on reflexively autocatalytic, food-generated (RAF) sets [22–25]. While focused primarily on networks with direct catalysis, where each chemical reaction requires a specific catalytic species, this body of work has yielded important insights including the potential for evolution in the absence of template-mediated replication [26,27]. Another promising non-genetic framework, the graded autocatalysis replication domain (GARD) model explored by Lancet and colleagues [28–31], considered assemblies of multiple chemical species that can change over time through mutually catalytic, non-covalent interactions. However, despite its virtues, the GARD model cannot readily be applied to metabolic networks and has not yet been framed with sufficient generality to aid us in understanding how chemical evolution might transition from non-genetic to genetic information encoding.

A potential general framework for describing adaptive change during origins of life lies in ecosystem ecology [32–34]. As discussed in the remainder of this paper, there is reason to suspect that by merging an ecological perspective with recent advances in understanding the properties and dynamics of chemical reaction networks [35–41] it may be possible to build a general, chemically agnostic theory for the origin of life. Such a theory would explain adaptive change independent of the existence of genetic polymers or cell-like encapsulation and could be used, therefore, to explore the possibility that genes and cells are products of gradual evolution. This would facilitate the side-by-side comparison of competing origin-of-life models and could guide the development of new experimental paradigms. Here, we integrate basic ecological and evolutionary concepts with chemical reaction network dynamics to provide a conceptual model for primordial evolution in spatially structured chemical ecosystems and the subsequent adaptive emergence of cellular individuation and genetic encoding.

## 3. The formal equivalence of biological and metabolic ecosystems

**Figure 1.**
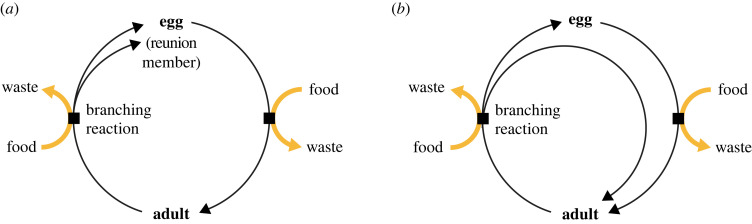
A biological life cycle shown to emphasize its autocatalytic features. Eggs and adults can be viewed as chemical complexes that are members of a simple autocatalytic cycle. This autocatalytic structure applies whether we focus on a single adult producing multiple eggs (*a*) or on an adult producing a single egg and then persisting after egg-production alongside its adult offspring (*b*).

A biological ecosystem comprises a location where food and energy flux allow the persistence of a community of one or more species. Each species in the community is composed of organisms whose life cycles are canonical examples of ACs. An AC can be defined as a series of reactions that consumes inputs, which we will call food, and includes reactions among a set of internal components, which we will call members, where there is at least one ‘branching reaction’ that confers stoichiometric growth [34,38]. Biological life cycles are simple examples of ACs whether we focus on a single adult producing at least two offspring or a long-lived adult that produces offspring over time (figure 1). The existence of branching reactions in these ACs permits sustained flow of energy and food to drive an increase in the count of organisms, which may be sufficient to offset organism loss by death or emigration.

When ecosystems contain more than one species, these species may be linked by diverse ecological interactions, including competition, predation and mutualism. These interspecific interactions can link autocatalytic life cycles together into higher-level ACs. A simple example would be a pair of obligately mutualistic species that exchange key resources. In that case, the exchanged resources are members of a combined AC, which contains at least two branching reactions (one in each life cycle). As a result, when we track all the chemical and physical resources, any real ecosystem forms a complicated chemical network with multiple interconnected and nested ACs. Thus, a biological ecosystem is a special case of an ACE: a localized environment that receives a flux of food and energy and sustains ACs.

Some chemicals produced by an ACE are not, formally, members of ACs. For example, waste products excreted by an organism will be generated whenever that species is alive, but the waste does not need to be present for the organism to reproduce. However, while at least four distinct roles can be assigned to elements of autocatalytic systems [40,42], such a classification is not needed here. All that matters for now is that ecosystems and subsets of ecosystems, including individual life cycles, use their component ACs to convert food and energy in the environment into more AC members.

Under this conception, an individual living cell (or organism) is also an ACE. Like a biological ecosystem, a cell contains a set of chemicals that collectively use food/energy from the environment to generate more of the same set of chemicals. The ability to make more of the cell's components, to self-propagate, underlies its capacity to replace components lost to the environment, to grow and to divide. Just like a biological ecosystem, the collective autocatalysis of a cell rests on numerous interconnected ACs.

**Figure 2.**
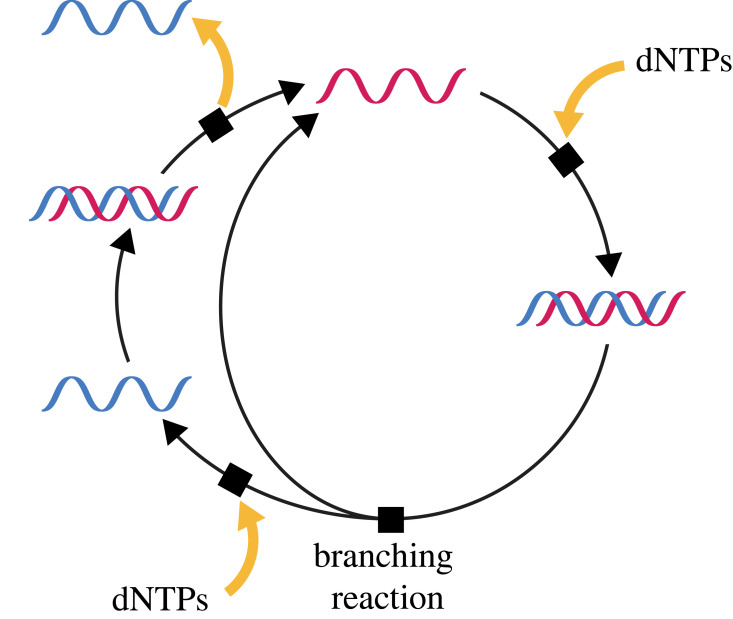
The gene life cycle of a modern cell. The figure has been drawn to emphasize the features of template-mediated replication that result in stoichiometric autocatalysis. As with biological or chemical ACs, the cycle is driven by a flux of food, in this case dNTPs, and may generate waste, in this case a copy of the complementary strand.

One important set of ACs inside a cell are those that result in gene replication (figure 2). Strand separation of a DNA duplex following second-strand synthesis is a branching reaction where each product can be converted back to a new duplex by complementary base pairing and new strand synthesis. Each stretch of DNA in a genome is, thus, a member of an AC. However, these genetic ACs cannot run in isolation but require enzymes (e.g. DNA polymerase) and building blocks (e.g. deoxy-ribonucleoside triphosphates) that, except in a test tube, are not provided for free by the external environment. Thus, a gene ‘life cycle’ is an obligate mutualist of other ACs within cellular metabolism.

Biochemists have long appreciated the existence of certain ACs within cellular metabolism, such as the Calvin or reductive citric acid cycles [43]. However, these named pathways represent only a tiny subset of the ACs hidden within cellular metabolism. Using recently developed principles for identifying stoichiometric ACs [38], analyses have identified huge numbers of overlapping ACs within metabolic networks [40]. A similar conclusion has also been reached using a non-stoichiometric definition of autocatalysis from RAF theory [44]. In principle, these metabolic ACs can serve as units of chemical memory or heritability similar to gene replication cycles [45].

**Figure 3.**
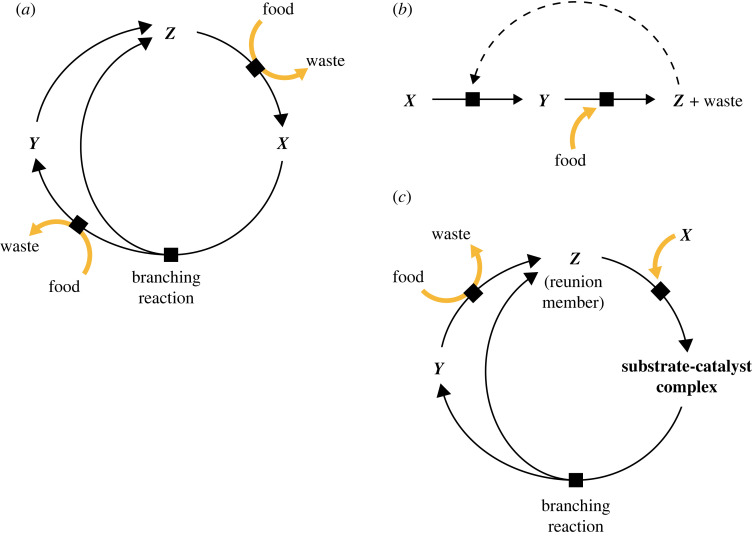
Autocatalytic cycles in chemical reaction networks. (*a*) A simple, stoichiometrically autocatalytic cycle. (*b*) An example of autocatalysis, where a catalyst, *Z*, promotes one of the reactions needed for its own synthesis. (*c*) The same cycle shown in *b*, redrawn to emphasize stoichiometric autocatalysis.

A full accounting of cellular ACs needs to consider that many enzymes catalyse reactions that promote, however indirectly, future protein synthesis. When a catalyst catalyses a reaction that supports its own synthesis, it is, by definition, an autocatalyst. Indeed, most functional enzymes in a cell are members of additional ACs that contribute to the ability of the cell to make more of its own components. This can be seen by redrawing the catalysed reaction to include the substrate-catalyst complex, whereupon it becomes clear that conversion of that complex into product-plus-catalyst functions as a branching reaction in an AC (figure 3*b,c*). This provides the basis for connecting RAF theory to other frameworks for exploring autocatalysis in chemical reaction networks.

The similarities between biological ecosystems and the chemical ecosystems of metabolically active cells go beyond the mere presence of ACs. The ACs in chemical ecosystems can show diverse pairwise interactions, such as competition, predation and mutualism, that are qualitatively and even quantitatively similar to those seen between species in biological ecosystems [34]. As one obvious example, each genetic AC is mutualistic with that of its complementary strand. The diversity of possible direct interactions between pairs of chemical ACs is greater than in conventional ecology, because there may be multiple chemical compounds associated with each AC, and each compound can be classified into one of four categories (food, waste, core member and non-core member [42]). However, while chemical networks may include interaction types that are not typically seen in conventional ecology, this is probably a matter of degree. Moreover, it is worth remembering that all ecosystems are chemical insofar as they are composed of the combined metabolisms of all their constituent organisms interacting with the chemistry of the physical environment.

Based on the preceding, we would argue that individual cells and localized biological ecosystems are both examples of ACEs. If cells are localized ecosystems, then it follows logically that populations of cells are analogous to meta-ecosystems, which is to say sets of semi-independent local ecosystems. Imagine seeding a Petri dish with one bacterial cell and coming back later to observe many cells on the plate, all derived by divisions of the first cell. Now imagine a local ecosystem that is surrounded by a set of similar, but empty, patches. Would one not expect the first ecosystem to have spread, via dispersal of its component organisms, into the surrounding patches? We will return later to the extent to which ecosystems have individual identity analogous to cells, but for now we ask the reader to accept a preliminary mapping of cells onto ecosystems and cellular populations onto meta-ecosystems.

## 4. The similarities of ecological succession and Darwinian evolution

Consider a population of organisms whose attributes come to change over a few generations. For such change to be considered ‘evolution,’ the differences accumulating over time need to be intrinsic, meaning that they are encoded in such a way that descendants tend to resemble their parents independent of the physical environment. This is what we mean when we say that evolution entails *heritable* changes in populations over time.

Can changes in biological ecosystems be intrinsic, or heritable? Organisms do not arise spontaneously from food. Rather, species life cycles are *seed-dependent*—they are not created by the physical (or external) environment but need to be triggered by the introduction of a ‘seed', which is to say a member of the AC, which in the case of species life cycles could be a fertile organism, a seed (in the narrow sense), a spore, or a vegetative propagule. In a chemical context, seeding can be accomplished by a member of the AC or a chemical/complex that can react to generate a member [40]. Seed-dependent autocatalytic systems have the property that, once seeded into a supportive ecosystem, they have the potential to persist indefinitely. In this sense, succession can, indeed, be seen as intrinsic change, because the set of species present at a moment in time is highly correlated with the set present at an earlier time, even if the physical environment changed greatly in between. Thus, seeding of species into an ecosystem represents a kind of ecological memory, which is analogous to the heritable changes that underlie evolution.

**Figure 4.**
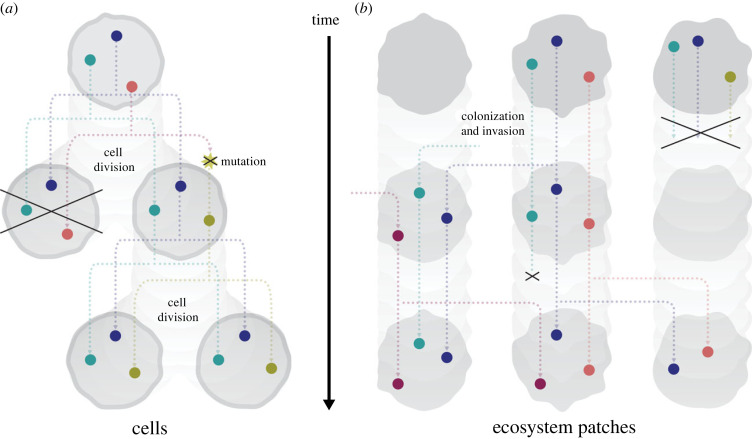
A comparison of cellular (*a*) and ecosystem evolution (*b*). In both cases, there are a certain number of cells/ecosystems at each point in time, characterized by a set of active ACs (coloured dots). ACs tend to persist through time, but over time the set of ACs present in the population/meta-ecosystem can change. In cells, heritability is (primarily) vertical, with new ACs arising via mutation, which entails extinction of a prior genetic AC and its replacement by a new one. In ecosystems, new ACs (species) can go extinct locally and can be acquired by dispersal from a nearby ecosystem or the broader species pool.

As ACE processes, succession via species seeding and evolution via genetic mutation have many deep similarities (figure 4). In succession, seeding of a new AC occurs via stochastic dispersal of a viable seed from a regional species pool. In evolution, mutation occurs when a rare chemical reaction yields a different DNA sequence (usually destroying an ancestral molecular sequence in the process) that is a member of a new gene replication AC. In either case, if the new AC is viable in that physical environment, it will become established. This will transition the ACE to the vicinity of a new dynamical attractor, resulting in a novel quasi-stable state where the new AC is active. An active AC is one that has been seeded and has not gone extinct, meaning that at least one member is currently present. Regardless of whether transitioning to the new attractor also entails extinction or altered flow through other ACs, seeding permits an ACE to adopt a new state, encoded in the set of active ACs, that could persist even if the physical environment changes.

Given the foregoing, how then is Darwinian evolution linked to succession or other kinds of intrinsic ecological change? Conventional Darwinian evolution occurs not at the level of the cell/organism but at the level of a population and entails changes in the frequency of gene replication ACs over time. It is important to remember, here, that within modern evolutionary biology a change in allele frequency is judged to be evolution regardless of the cause of that change. Thus, evolution can be stochastic (genetic drift) or result from the effects of ACs on cell survival and reproduction (selection).

If a single cell is equated with a localized chemical ecosystem, then a population of cells is equivalent to an autocatalytic chemical meta-ecosystem or ACME (figure 4). The ecological analogue of evolution applies when the frequency of different ecosystem compositions (where an ecosystem's composition is its set of active ACs) changes in an ACME. Such a change in ecosystem composition could happen by chance, analogous to genetic drift, for example when disturbance randomly extinguishes some ecosystem compositions. However, change could also be driven by intrinsic differences between ecosystems (subcommunities, *sensu* [46]), for example when some ecosystem compositions are more resistant to disturbance and/or better at colonizing new environmental patches than others.

Based on this analysis, changes in biological meta-ecosystems and genetic populations are deeply homologous: in both cases, changes in the abundance of different ACs in an ACME provide the basis of intrinsic (thus heritable) change. Moreover, in each context, the spreading of new ACE compositions could be due to either chance (drift) or the effects of ACs on ACE fitness (selection). We will return to the differences between ecological and evolutionary changes, but first let us connect this insight to the origin-of-life problem.

## 5. The origin of life as the emergence of an autocatalytic chemical ecosystem process

Even non-biological chemical reaction networks are replete with ACs [40,47], including simple stoichiometric ACs (figure 3*a*) and cases of catalysts that catalyse steps in their own synthesis (figure 3*b,c*). Additionally, it is plausible that abiotic ACEs can generate polymers like RNA that have some tendency towards template-mediated polymerization or ligation, in which case gene-like ACs (figure 2) might also be present. As a result, because the Earth was formed and remains out of thermodynamic equilibrium (due to energy influx from the Sun and disequilibria established during solar system formation), many potential ACs would have been viable somewhere on the planet.

Because the Earth is large and heterogeneous, there was surely enough spatial structure to ensure that localized ACEs would have the potential to differ, implying that, from the very beginning, there must have been ACMEs at various spatial scales on Earth. Examples of possible primordial ACMEs include large, two-dimensional mineral surfaces, discontinuous mineral patches on the ocean floor, or networks of pores in a rocky matrix. Within such meta-ecosystems, succession-like dynamics would be expected, due to successive seeding of ACs, whether by import events from distant sites (including delivery from space) or by the local occurrence of rare (i.e. very slow) chemical reactions. Despite lacking biological entities like cells, a prebiotic succession process, like conventional ecology, would have been constrained by the interactions among ACs. For example, analyses of real chemical reaction networks, with a set of food species defined, showed there are limits on the order in which ACs can be seeded: whereas some seeds could establish ACs given only food flux (analogous to primary producers), other ACs could only be seeded later in succession, after all of its food species had become available (analogous to consumers) [40].

Taken together, it seems inevitable that chemical ecosystems on the prebiotic Earth would have complexified by a process resembling biological succession with chemical ACs providing the units of heritable change. Combined with the insight that ecological change and Darwinian evolution are both examples of ACE processes, it is likely that the first prebiotic systems showed succession-like dynamics but became more and more paradigmatically Darwinian in character over time. To map out such a transition we need to confront two key differences between ecological succession (whether at the biological or chemical level) and biological evolution. We will address these salient differences in the next two sections before summarizing our argument and proposing avenues for further research.

## 6. Cells and autopoiesis: the emergence of self

Evolving populations are composed of entities (cells or organisms) that are *autopoietic* [48], meaning they define and maintain their own boundaries. Autopoiesis allows living entities to grow and then divide into separate units that each have the ingredients needed to persist, grow and divide again. By contrast, even if ecosystems are spatially discrete, their boundaries are frequently tied to discontinuities in the external environment rather than being endogenous. As a result, ecosystems do not usually undergo anything resembling a growth-division life cycle.

Within the meta-ecosystem framework developed here, the key distinction between conventional ecosystems and cells/organisms is not about autopoiesis *per se* but about the independence of AC dispersal. In a growth-division life cycle, all the ACs in a parent ACE co-disperse to each descendent ACE. Co-dispersal occurs when a daughter ACE receives at least one member molecule (e.g. a single chromosome) of multiple ACs present in the parent, which ensures that those ACs will each be activated in the daughter ACE. Co-dispersal aligns the interests of all ACs, favouring cooperation for the common good and the suppression of cheaters [49–51]. In perfect co-dispersal, if ecosystem X receives members of one AC from ‘parent’ ecosystem Y, then it will also receive members of all other ACs from Y. In contrast, the species ACs of biological ecosystems typically show independent dispersal, where the ACs in parent ecosystems assort independently to ‘offspring’ ecosystems. Independent dispersal makes it easier for selfish species, for example swarming locusts, to persist in a meta-ecosystem even if they exploit resources in each local ecosystem in a way that undermines the persistence of those local ecosystems. If the first evolving systems resembled canonical meta-ecosystems with independent AC dispersal, then explaining the origin of autopoietic cells requires that we provide a mechanism by which co-dispersal dynamics could arise—ideally a mechanism that predicts a selective advantage to those ACs whose members promote co-dispersal.

Before we explore selection for co-dispersal, it is worth noting that the claim that all ACs in living cells/organisms co-disperse is oversimplified [52]. Growth-division life cycles may be complex, especially for multi-cellular entities [53,54], which can include deviations from strict AC co-dispersal. For example, sexual syngamy and horizontal gene transfer allow gene life cycles to disperse independently, opening the door for selfish genetic elements to arise and persist. Conversely, traditional ecosystems cannot always be equated with assemblages of independently dispersing organisms. For example, certain pairs of species have evolved mechanisms that promote co-dispersal, as illustrated by certain lichen-forming fungi which produce structures (soredia) that allow for co-dispersal with associated algae.

Clements [55] famously argued in 1916 that communities are individuals with emergent properties like fitness. He focused not on physical co-dispersal *per se* but on the capacity of one species to modify the ecosystem to enable certain other species to establish and co-occur. Most ecologists have rejected Clements' view, most famously Gleason [56] and Whittaker [57], and instead emphasized competitive dynamics and independent dispersal. In microbial ecology, however, there is increasing acceptance of the possibility of community individuation and the existence of co-dispersing consortia (e.g. lichens, biofilms) that blur the distinctions between ecosystems and individuals [58,59].

In the origins-of-life field, it remains controversial whether a growth-division life cycle was present from the start or was acquired later. Several prominent theories posit the spontaneous emergence of self-organizing, self-replicating structures, for example coacervates droplets [60,61] organized by electrostatic charges, micelles [29,31,62] organized by a hydrophobic/hydrophilic phase separation, or protocells [63,64] organized by bounding membranes. Others have argued that such autopoietic entities are too complicated to arise spontaneously, a position we are inclined towards. In that case, a growth-division life cycle must be an evolved feature regardless of whether the first evolving entities were bounded by discontinuities in the physical environment or lacked boundaries entirely, for example ACEs confined to a continuous mineral surface [15,65].

Under the view that autopoiesis is an evolved characteristic, the origin of the first autopoietic system was the first major transition in evolution [54,66]. This transition would have entailed differential survival of ACs as a function of their effect on the ability of ecosystem patches to persist and export their components to other ecosystem patches. There is evidence from ecology that selection can act at the community/ecosystem level in spatially structured meta-ecosystems [46,67] and that this can scaffold the emergence of new levels of selection [68]. By extrapolation, we may imagine spatially structured prebiotic chemical ecosystems where some ACs reduced the risk of local extinction. In such a situation, there are likely to be disturbance regimes under which ACs can become enriched when their members co-disperse with ACs that enhance ACE fitness. Thus, ACs whose members promote co-dispersal, for example by chemical tethering or entrapping multiple chemical seeds, could be favoured by ecosystem-level selection. Combined with the capacity for amphiphilic compounds to self-assemble into vesicles, it is not hard to imagine how a protocell-like compound seed (i.e. a unit seeding many ACs at once) could arise and eventually give rise to a protocell [13]. Thus, recognizing the formal similarities of localized ecosystems and autopoietic life provides a framework for understanding how an ACE process could transition from individualistic AC dispersal to co-dispersal and, ultimately, to a growth-division life cycle.

## 7. Genes and digital information: origins of information encoding

Living cells/organisms have genetic systems, which differ in many ways from either biological ecosystems or ACEs that could plausibly originate spontaneously on the early Earth. Of these differences, we will highlight two characteristics of modern life that together explain its capacity for efficient adaptive evolution: (i) catalytic heteropolymers with template-mediated replication and (ii) chemically stable genomes with information encoded in covalent bonds between adjacent bases. For this discussion we will contrast modern genetic cells with autopoietic ACEs, such as protocells, that have growth-division life cycles. However, this does not mean that we reject the possibility that some aspects of genetic encoding, for example use of templating polymers, could have pre-dated autopoiesis.

New ACs in prebiotic chemical ecosystems would typically be triggered by import of a seed molecule, analogous to ecological succession via dispersal from a broader regional pool. Such a mechanism seeds ACs that are uncorrelated with pre-existing ACs. As a result, there is a relatively high probability that a new AC will be inviable or, if it is viable, that it causes a drastic change in emergent phenotypes of the local ecosystem. In contrast, nucleic acid molecules have a generic ability to undergo template-mediated replication, independent of the cellular functions of those sequences: almost any DNA sequence is a member of an AC (along with its reverse-complementary sequence's AC). Moreover, the seeding of a new gene replication AC happens via mutation, where a rare chemical reaction replaces a pre-existing sequence with a new, but very similar mutant sequence. Genetic mutation is, thus, a seeding process where the resulting seed is highly correlated to the prior state. This increases the chances that a new AC will be viable and explains the ability of genetic populations to constantly tinker with already-functional phenotypes, permitting the systematic exploration of adaptive landscapes by hill-climbing.

Despite the great differences in the evolutionary dynamics of genetic and non-genetic ACEs, it is not hard to understand how genetic systems could arise. Remember that new ACs can be triggered either by seeding from the outside *or* by rare reactions among species already present. Novel reactions among pre-existing functional groups within a prebiotic ACE will tend to generate new chemicals that resemble pre-existing chemicals. Moreover, paradigmatic Darwinian evolution also entails genetic variation introduced by migration or lateral gene transfer. In this way, the distinction between genetic mutations and ecological seeding becomes blurred. Moreover, there are mechanisms available that could result in a system becoming progressively more genetic (and less ecological) over time.

The chemistry of the prebiotic Earth implies that a population of non-genetic protocells probably produced the building blocks of RNA (and/or other catalytic polymers capable of template-mediated replication) [69,70]. Random polymerization of the resultant monomers would yield diverse sequences, some of which might enhance the capacity of protocells to grow and divide by conferring ecosystem services such as enhancing stability (e.g. protection from osmotic shock) or growth rate (for example by catalysing metabolic processes or catalysing the production of other classes of polymers, such as peptides). Those protocells with more functional polymers will be more productive and, due to template-mediated replication, will tend to have descendent protocells that are enriched for the same (or similar) functional polymers. This means that selection at the ecosystem (=protocell) fitness can yield more functional polymer assemblages over time [9].

Given a finite availability of monomer precursors, selection on protocell fitness will also indirectly select for polymers that enhance genetic feedback. Sequences that catalyse polymerization or enhance the fidelity of template-mediated polymerization would tend to enhance protocell fitness by directing monomers more efficiently towards functional polymers. Thus, analogous to the evolution of lower mutation rates over time, protocell populations will tend to accumulate polymers that promote more effective genetic inheritance mechanisms. It has been shown that ecological interactions among different kinds of protocells within a meta-ecosystem (=population) can favour the persistence of protocells with genetic inheritance [71]. As a result, starting from slow and error-prone non-enzymatic RNA polymerization, protocell-level selection could gradually assemble sets of RNAs that collectively achieve efficient and accurate template-mediated replication. In principle, selection would also enrich for ACs that promoted the controlled synthesis of functional peptides, thus providing a pathway towards ribosomal translation.

The transition of protocells from ecosystems of small-molecule ACs to ecosystems that also include cooperating, template-replicating polymers does not require a genome or the coordination of cellular and genomic replication. Even without genomes, offspring protocells could resemble their parents due to analogue or compositional inheritance [72,73], where ACs in parent ACEs have enough members that, by random segregation, all offspring ACEs have a full complement of ACs. The origin of the genome can be viewed as a transition to digital inheritance [74], where information comes to be locked in the covalent bonds of a single chromosome [75]. Combined with mechanisms for ensuring that each daughter cell receives exactly one copy of each chromosome, the consolidation of a genome allows for reliable inheritance even when there are very large numbers of ACs and insufficient space for all of them to be represented by enough member molecules that both offspring cells reliably receive the full complement. Mathematical models have demonstrated that given a protocell with analogue inheritance involving many RNA molecules, a single master genome can gradually evolve [76,77].

In this section, we have shown that two key features of modern cells, template-replicating heteropolymers and covalent genomes enhance cellular adaptation, the former by allowing systems to build on prior success, the latter by reducing noise and permitting effective responses to smaller selection coefficients. We have also provided logical arguments to support the claim that both features could gradually evolve in populations of compositional protocells. Combined with our prior discussions, this insight implies that paradigmatic Darwinian evolution is a derived rather than ancestral feature of life—a claim that has profound implications for the study of life's origin on Earth and (presumably) elsewhere in the Universe.

## 8. Implications of eco-evolutionary continuity

In this paper, we have argued that cells and organisms are ecosystems with a formal equivalence to local patches in a biological or strictly chemical meta-ecosystem. While modern cells are self-bounding and contain many ACs that include genetic polymers, these are differences in degree rather than kind. This observation means that autocatalytic ecology not only defines formal identity of very different kinds of ACEs, but also provides a framework for exploring the gradual evolution of genetic cells from non-genetic, non-autopoietic ancestors.

The position staked out here is that ecological and evolutionary changes are not inherently different but represent ends of a continuum. This implies that it ought to be possible to articulate a general theory of change that applies to phenomena as diverse as succession, development, Darwinian evolution and chemical reaction dynamics. Indeed, steps towards such overarching theory have been developed under the rubric of stochastic population processes [78]. Such a blurring of the evolution-ecology distinction supports the claim that food-driven chemical reaction networks change similarly to biological ecosystems, allowing new ways to conceptualize the origin of life. This new framework, chemical ecosystem ecology [34,40,79], suggests that the origin of life is best understood as a process whereby ACMEs change adaptively prior to the onset of Darwinian evolution, resulting in the acquisition of autopoiesis and genetic encoding. Such an insight may allow for the development of new formal theories of abiogenesis that avoid invoking the spontaneous emergence of complex genetic systems. Additionally, early iterations of this ecology-first perspective have already stimulated new kinds of laboratory experiment [79,80]. Thus, by bringing together expertise in ecology, evolution and autocatalytic chemistry, it may prove possible one day to document the *de novo* appearance of adaptively evolving chemical systems—systems that would go a long way towards blurring the supposed sharp boundary between life and non-life.

## Data Availability

This article has no additional data.
